# Current clinical practice for familial adenomatous polyposis in Japan: A nationwide multicenter study

**DOI:** 10.1002/ags3.12577

**Published:** 2022-05-24

**Authors:** Takaaki Matsubara, Naohito Beppu, Masataka Ikeda, Hideyuki Ishida, Yoji Takeuchi, Toshiya Nagasaki, Akinari Takao, Kazuhito Sasaki, Kiwamu Akagi, Tomoya Sudo, Hideki Ueno, Tatsuro Yamaguchi, Naohiro Tomita, Yoichi Ajioka

**Affiliations:** ^1^ Department of Surgery, Division of Lower Gastrointestinal Surgery Hyogo College of Medicine Nishinomiya Hyogo Japan; ^2^ Study Group for Familial Adenomatous Polyposis in the Japanese Society for Cancer of the Colon and Rectum Tokyo Japan; ^3^ Department of Digestive Tract and General surgery, Saitama Medical Center Saitama Medical University Saitama Japan; ^4^ Department of Gastrointestinal Oncology and Department of Genetic Oncology, Department of Hereditary Tumors Osaka International Cancer Institute Osaka Japan; ^5^ Department of Gastroenterological Surgery Cancer Institute Hospital of the Japanese Foundation for Cancer Research Tokyo Japan; ^6^ Department of Gastroenterology Tokyo Metropolitan Cancer and Infectious Diseases Center Komagome Hospital Tokyo Japan; ^7^ Department of Surgical Oncology, Faculty of Medicine The University of Tokyo Tokyo Japan; ^8^ Division of Molecular Diagnosis and Cancer Prevention Saitama Cancer Center Saitama Japan; ^9^ Department of Surgery Kurume University School of Medicine Kurume Japan; ^10^ Department of Surgery National Defense Medical College Saitama Japan; ^11^ Department of Clinical Genetics Tokyo Metropolitan Cancer and Infectious Diseases Center Komagome Hospital Tokyo Japan; ^12^ Cancer Treatment Center Toyonaka Municipal Hospital Osaka Japan; ^13^ Division of Molecular and Diagnostic Pathology Niigata University Graduate School of Medical and Dental Sciences Niigata Japan

**Keywords:** colorectal cancer, desmoid tumors, familial adenomatous polyposis, non‐colectomy

## Abstract

**Introduction:**

In Japanese patients with familial adenomatous polyposis (FAP), colectomy tends to be postponed or avoided.

**Aim:**

This study aimed to clarify the current clinical practice from a Japanese multicenter cohort study database.

**Methods:**

We analyzed the records of 250 patients with non‐dense FAP who did not require colorectal cancer removal. The clinical outcomes were compared between patients who received colectomy (n = 142) (Group A) and those who did not receive colectomy (n = 108) (Group B).

**Results:**

The colectomy rate based on the age at the final follow‐up examination was 46%, 60%, 54%, 65%, at ≤29, 30–39, 40–49, and ≥ 50 years, respectively (*P* = .11). The development of colorectal cancer did not differ between Groups A and B (25% vs 22% *P* = .67); however, colorectal cancer was diagnosed at the Tis stage in 88% of the patients with colorectal cancer in Group B, and 34% of the patients with colorectal cancer in Group A (*P* < .01). Regarding survival, all patients in Group B were alive at the final follow‐up examination. In contrast, six patients in Group A died, including three patients with desmoid tumors and one with colon cancer.

**Conclusion:**

Over one‐third of patients with non‐dense FAP (polyps ≤ 1000) in Japan did not receive colectomy at >30 years of age, and patients who managed without colectomy showed acceptable survival with the early diagnosis of colorectal cancer, and a very low incidence of desmoid tumor development, indicating that this approach represents a potential option for the management of selected non‐dense FAP patients.

## Graphical abstract

Over one‐third of patients with non‐dense FAP in Japan did not receive colectomy at >30 years of age, and patients managed without colectomy showed acceptable survival with the early diagnosis of colorectal cancer, and a very low incidence of desmoid tumor development, indicating that this approach represents a potential option for the management of selected non‐dense FAP patients.

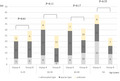

## 1 INTRODUCTION

Approximately 150 000 people are affected by colorectal cancer (CRC) annually, making it the most common type of cancer in Japan.^1^ Approximately 20%‐30% of CRCs are potentially linked to genetic factors, and hereditary CRC syndromes account for 3%‐5% of all CRCs.^2^ Familial adenomatous polyposis (FAP) is an autosomal dominant genetic disorder caused by a germline variant of the adenomatous polyposis coli (APC) gene; the main symptom is the development of multiple adenomas in the colon.^3^ The causative gene is the APC gene on chromosome 5; the frequency in Japan is one in 17 400.^4^, ^5^

In the natural history of FAP, the incidence of CRC is 50% in patients in their 40s and 100% in patients in their 50s.^6^ Thus, colonoscopy is recommended from 10 years of age and patients with profuse and sparse FAP are recommended to undergo examinations every 1‐2 years, while those with attenuated type are recommended to undergo examinations every 2‐3 years.^7^ The Japanese Society for Cancer of the Colon and Rectum (JSCCR) Clinical Practice Guidelines for Hereditary Colorectal Cancer recommend that FAP patients undergo total proctocolectomy in their 20s.^7^ However, after total colectomy, the quality of life (QOL) is significantly decreased due to bowel dysfunction and dehydration. Furthermore, total proctocolectomy induces the risk of bowel obstruction and desmoid tumor development. Notably, desmoid tumors are the second leading cause of death in patients with FAP.^8^, ^9^, ^10^ On the other hand, the safety of polypectomy by colonoscopy has increased with the development of equipment and techniques. Thus, in order to avoid the complications associated with total colectomy, an alternative approach has been proposed with colectomy with preservation of the rectum, and surveillance of the rectum by colonoscopy for the lifetime of the patient.^11^, ^12^ This management is associated with good QOL, without compromising survival. In accordance with these concepts, clinical attempts have been made to delay or avoid colectomy by performing intensive follow‐up by colonoscopy for patients with sparse and attenuated type FAP.^13^, ^14^

Consequently, the management of FAP have been diversifying in Japan. Thus, the present study aimed to clarify the current clinical practice for sparse and attenuated type of FAP in Japan using a Japanese retrospective multicenter cohort study database.

## 2 METHODS

### 2.1 Data source

This study included Japanese FAP patients who were listed in the database of the working group of the Japanese Society for Cancer of the Colon and Rectum (JSCCR) “multicenter retrospective study of FAP patients.” These data were retrospectively collected from 35 JSCCR member institutions, which are leading hospitals for colorectal treatment. The included patients were diagnosed with FAP before 2018. The diagnostic criteria for FAP included a clinical or genetic diagnosis of FAP. The criteria for the clinical diagnosis of FAP were as follows: (1) approximately ≥100 adenomas in the colorectum, regardless of family history; (2) number of adenomas <100; however, patient has a family history of FAP.^7^ A genetic diagnosis was conducted for patients with germline mutations in the APC gene.

The surgical timing, follow‐up methods, and surveillance for FAP patients were decided by each institution and each physician. The present study was approved by the ethical committees of the JSCCR (90‐5) and Hyogo College of Medicine (3943).

### 2.2 Patients

**FIGURE 1:**
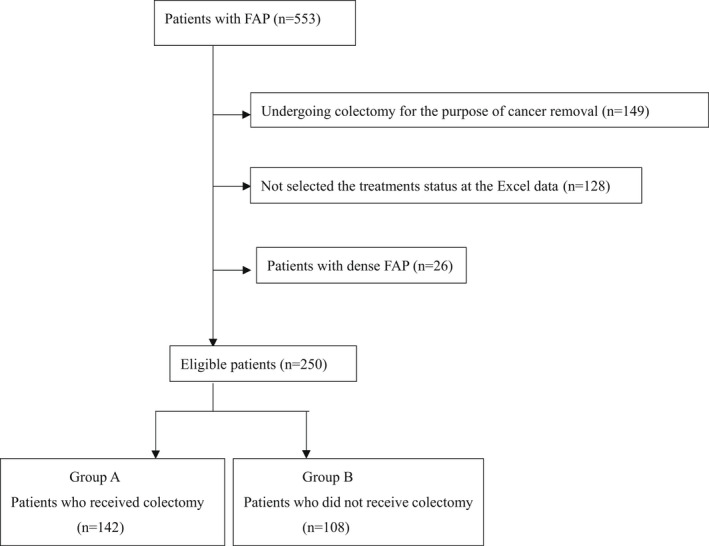
Flowchart of the included patients. FAP, familial adenomatous polyposis; Group a, patients who received colectomy; Group B, patients who did not receive colectomy

This database included 553 FAP patients. The following patients were excluded: patients undergoing proctocolectomy for the purpose of cancer removal (n = 149), patients with missing data, patients who are not selected for the treatments status at the Excel data (received colectomy or not received colectomy) (n = 128), and patients with dense FAP (n = 26). Thus, a total of 250 patients were included in this study (Figure 1). The characteristics of patients who received colectomy (n = 142) (Group A) and those who did not receive colectomy (n = 108) (Group B) were compared. The surgical procedure of Group A was ileorectal anastomosis (IRA), n = 68; hand‐sewn ileal pouch anal anastomosis (IPAA), n = 38; stapled IPAA, n = 22; partial colectomy, n = 5; total colectomy and permanent ileostomy, n = 4; and unknown and others; n = 5.

This study investigated the following data: sex, age at FAP diagnosis, follow‐up period, follow‐up status, polyp density, genetic testing, and detection of pathogenic APC variant. The follow‐up status was classified as lost to follow‐up and followed, and patients who did not visit the hospital for 2 years were considered lost to follow‐up. Polyp density was classified into three categories as follows: dense type, ≥1001 colorectal polyps; 100‐1000 colorectal polyps, sparse type; and ≤99 colorectal polyps, attenuated type.^15^

**FIGURE 2:**
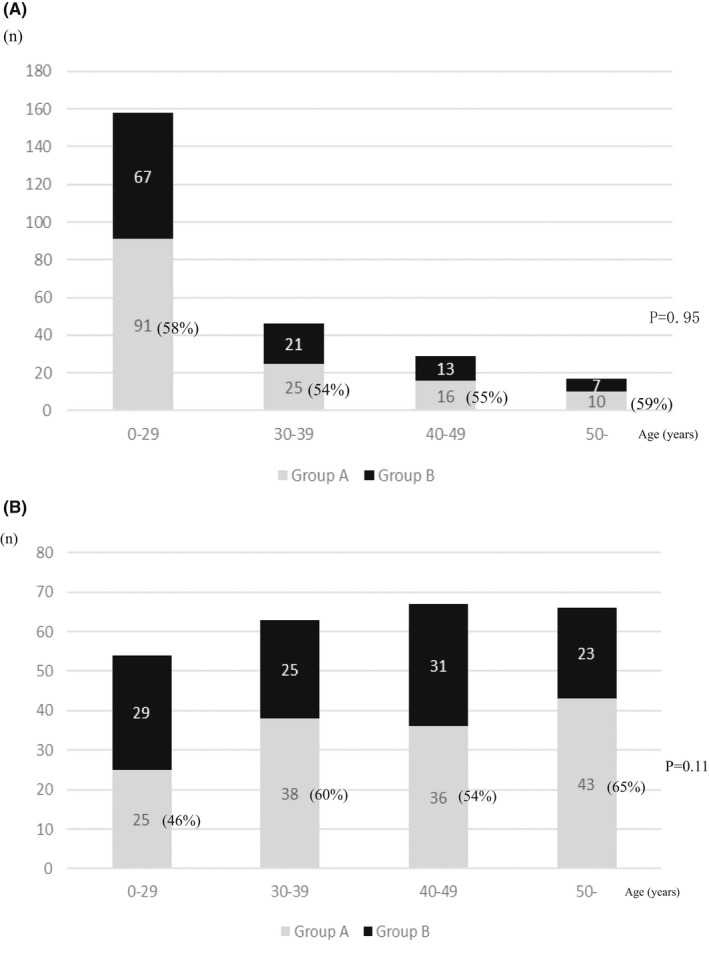
(A) Rate of patients managed with and without colectomy according to the age at the diagnosis of FAP. FAP, familial adenomatous polyposis; Group A, patients who received colectomy; Group B, patients who did not receive colectomy. (B) Rate of patients managed with and without colectomy according to the age at the final follow‐up examination. Group A, patients who received colectomy; Group B, patients who did not receive colectomy

**FIGURE 3:**
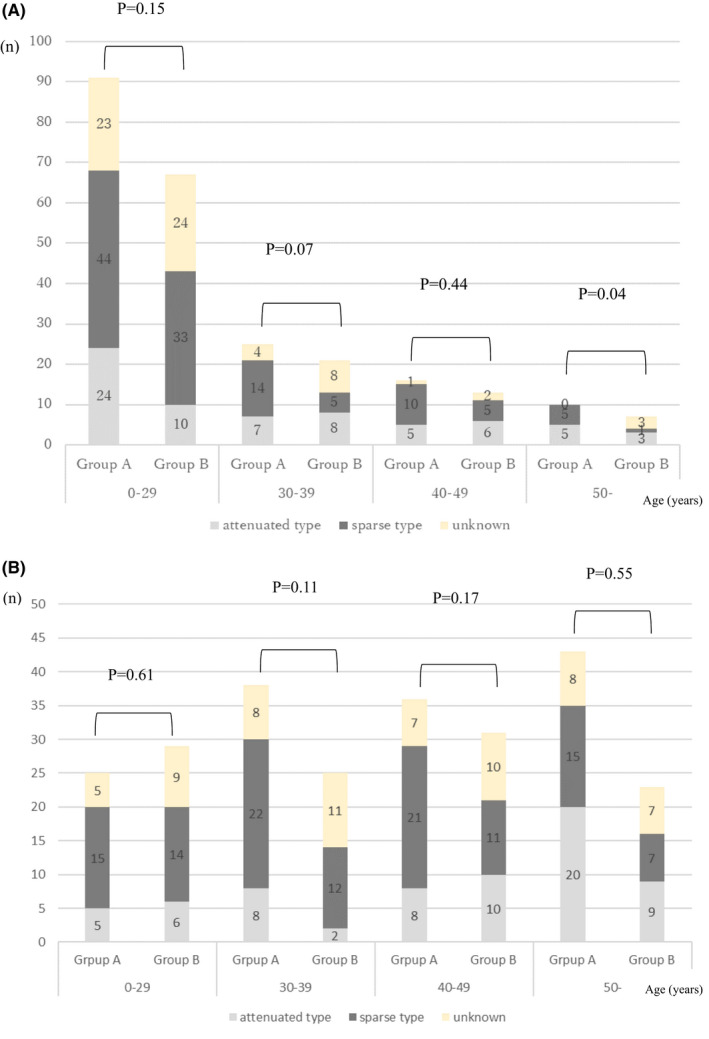
(A) Polyp density in patients managed with and without colectomy according to the age at the diagnosis of FAP. FAP, familial adenomatous polyposis; Group A, patients who received colectomy; Group B, patients who did not receive colectomy. (B) Polyp density in patients managed with and without colectomy according to the age at the final follow‐up examination. Group A, patients who received colectomy; Group B, patients who did not receive colectomy

The rates of patients managed with and without colectomy according to the age at the diagnosis of FAP and the age at the final follow‐up examination are shown in Figures 2a and b, respectively. In addition, the polyp density (sparse and attenuated type) in Groups A and B according to the age at the diagnosis of FAP and the age at the final follow‐up examination is shown in Figures 3a and b, respectively. In the graphs, the age groups were ≤29, 30‐39, 40‐49, and ≥50 years.

With regard to concomitant lesions in patients with FAP, duodenal adenoma and its Spigelman classification, papillary adenoma, fundic gland polyp, gastric adenoma, gastric cancer, desmoid tumors (intraperitoneal, abdominal wall), mandibular tumor, thyroid tumor, brain tumor, adrenal tumor, and colorectal cancer were compared between the two groups.

Regarding desmoid tumors, patients for whom a tumor site (intraperitoneal, abdominal wall) was recorded in the database were counted. In Group A, colorectal cancer cases included patients who were diagnosed by colonoscopy during the follow‐up period in addition and those diagnosed based on the examination of a surgical specimen at colectomy. On the other hand, in Group B, colorectal cancer cases included patients who were diagnosed by colonoscopy during the follow‐up period. Furthermore, colorectal cancer was classified according to the pathological T factor (Tis vs T1 or deeper).

### 2.3 Statistical analysis

Descriptive analyses are presented as the mean ± standard deviation or the median (range) for quantitative data and as the number of cases (percentage of cases) for categorical variables. Student's *t‐*test or the Mann‐Whitney U test were used for the comparison of quantitative data, while the chi‐squared test or, where appropriate, Fisher’s exact probability test were used for the comparison of categorical variables.

Survival curves were generated using the Kaplan‐Meier method and the log‐rank test was used to compare survival. All statistical analyses were performed using the JMP software program (version 14.0, SAS Institute). *P* values of <.05 were considered to indicate statistical significance.

## 3 RESULTS

### 3.1 Patient characteristics

The characteristics of the patients in Groups A (n = 142) and B (n = 108) are shown in Table 1. There were no significant differences between the two groups with regard to sex, age at the diagnosis of FAP, follow‐up period after the diagnosis of FAP, polyp density, genetic testing, or detection of pathogenic APC variant. On the other hand, a significantly higher percentage of patients in Group A were lost to follow‐up in comparison to Group B (34% vs 20%, *P* = .003).

**TABLE 1:** Patient characteristics

	Group A n = 142	Group B n = 108	*P* value
Sex			
Male	65 (46)	43 (40)	.35
Female	77 (54)	65 (60)	
Median age at the diagnosis of FAP (years)	26 (range; 0‐73)	24 (range; 0‐71)	.49
Median follow‐up period after diagnosis (years)	11 (range; 0‐77)	13 (range; 0‐73)	.14
Follow‐up status			
Lost to follow‐up	48 (34)	22 (20)	.003
Followed	87 (61)	86 (80)	
Dead	6 (4)	0 (0)	
Unknown	1 (1)	0 (0)	
Polyp density			
Sparse	73 (51)	44 (41)	.78
Attenuated	41 (29)	27 (25)	
Unknown	28 (20)	37(34)	
Genetic testing			
Yes	58 (41)	37 (34)	.29
No	84 (59)	71 (66)	
Detection of pathogenic APC variant			
Yes	50 (86)	28 (76)	.13
No	6 (10)	8 (22)	
Unknown	2 (3)	1 (3)	

*Note:* Group A, patients who received colectomy; Group B, patients who did not receive colectomy.

Abbreviation: APC, adenomatous polyposis coli.

### 3.2 Colectomy rate

The colectomy rate according to the age at the diagnosis of FAP was 58%, 54%, 55%, and 59% in patients of ≤29, 30‐39, 40‐49, and ≥50 years of age, respectively (*P* = 0.95) (Figure 2a). The colectomy rate according to the age of at the final follow‐up examination was 46%, 60%, 54%, 65% in patients of ≤29, 30‐39, 40‐49, and ≥50 years of age, respectively (*P* = .11; Figure 2b).

### 3.3 Polyp density in groups A and B

The polyp density in Groups A and B according to the age at the diagnosis of FAP is shown in Figure 3a. The distribution of sparse type and attenuated type did not differ to a statistically significant extent in any of the age groups except for the ≥50 years group (≤29 years, *P* = .15; 30‐39 years, *P* = .07; 40‐49 years, *P* = .44; ≥50 years, *P* = .04). However, the number of patients of ≥50 years of age was limited (Group A, n = 10, Group B, n = 7), and the significance was limited. Furthermore, the polyp density was compared between Groups A and B according to the age at the final follow‐up examination (Figure 3b). The distribution of sparse type and attenuated type did not differ to a statistically significant extent in any age group (≤29 years, *P* = .61; 30‐39 years, *P* = .11; 40–49 years, *P* = .17; ≥50 years, *P* = .55).

### 3.4 Concomitant lesions in patients with FAP

Table 2 shows the concomitant lesions in patients with FAP in Groups A and B. The incidence of desmoid tumors in Group A was significantly higher than that in Group B (10% vs 1%, *P* < .01). Although the rate of colorectal cancer did not differ between the two groups (25% vs 22% *P* = .67), the Tis stage at the diagnosis was significantly higher in Group B than in Group A (34% vs 88%, *P* < .01). With regard to other tumors, the incidence of papillary adenoma was significantly higher in Group A than in Group B (18% vs 7%, *P* = .01), while the incidence of gastric adenoma was significantly higher in Group B than in Group A (20% vs 43%, *P* < .01). There were no significant differences between the two groups in the incidence rates of adenoma of the duodenum, fundic gland polyp, gastric cancer, mandibular tumor, thyroid tumor, brain tumor, or adrenal tumor.

**TABLE 2:** Concomitant lesions in patients with FAP

	Group A n = 142 (%)	Group B n = 108 (%)	*P* value
Adenoma of duodenum			
Yes	80 (56)	65 (60)	.70
No	40 (28)	29 (27)	
Unknown	22 (16)	14 (13)	
Spigelman classification			
Grade 1	18 (23)	14 (22)	.20
Grade 2	16 (20)	10 (15)	
Grade 3	10 (13)	19 (29)	
Grade 4	9 (11)	8 (12)	
Unknown	27 (34)	14 (22)	
Papillary adenoma			
Yes	26 (18)	8 (7)	.01
No	80 (56)	76 (70)	
Unknown	36 (25)	24 (22)	
Fundic gland polyposis			
Yes	84 (59)	62 (57)	.75
No	42 (30)	34 (32)	
Unknown	16 (11)	12 (11)	
Gastric adenomas			
Yes	28 (20)	46 (43)	<.01
No	94 (66)	49 (45)	
Unknown	20 (14)	13 (12)	
Gastric cancer			
Yes	20 (14)	12 (11)	.46
No	105 (74)	84 (78)	
Unknown	17 (12)	12 (11)	
Desmoid tumor			
Yes	14 (10)	1 (1)	<.01
No	104 (73)	80 (74)	
Unknown	24 (17)	27 (25)	
Desmoid tumor location			
Abdominal wall	2 (14)	0 (0)	.37
Intra‐abdominal	12 (86)	1 (100)	
Mandibular tumor			
Yes	5 (3)	4 (4)	.97
No	99 (70)	81 (75)	
Unknown	38 (27)	23 (21)	
Thyroid tumors			
Yes	8 (6)	6 (6)	.82
No	110 (77)	80 (74)	
Unknown	24 (17)	22 (20)	
Brain tumor			
Yes	1 (1)	0 (0)	.82
No	67 (47)	43 (40)	
Unknown	74 (52)	65 (60)	
Adrenal tumor			
Yes	2 (1)	4 (4)	.23
No	116 (82)	60 (55)	
Unknown	24 (17)	44 (41)	
Diagnosis of colorectal cancer			
Presence	35 (25)	24 (22)	.67
Absence	104 (73)	81 (75)	
Unknown	3 (2)	3 (3)	
Opportunity for the diagnosis of colorectal cancer (Group A: n = 35, B: n = 24)			
Colonoscopy	9 (26)	24 (100)	<.01
Surgical specimen	26 (74)	0 (0)	
Depth of colorectal cancer			
Tis	12 (34)	21 (88)	<.01
T1 or deeper	15 (43)	1 (4)	
Unknown	8 (23)	2 (8)	

*Note:* Group A, patients who received colectomy; Group B, patients who did not receive colectomy; Group A, patients who received colectomy; Group B, patients who did not receive colectomy.

Abbreviation: FAP, familial adenomatous polyposis.

### 3.5 Overall survival

**FIGURE 4:**
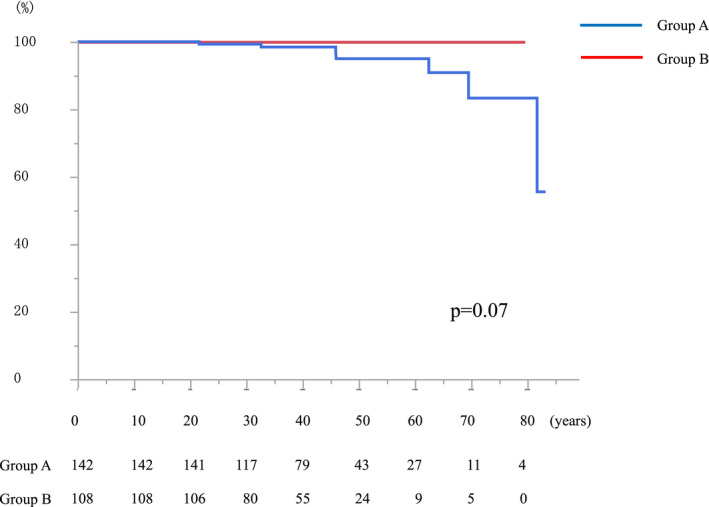
Overall survival. Group A, patients who received colectomy; Group B, patients who did not receive colectomy

Figure 4 shows the overall survival of the two groups. The median patient age was 40 years (9‐83 years). Six patients in Group A died (cause of death: desmoid tumor, n = 3; colon cancer, n = 1; gastric cancer, n = 1; sepsis, n = 1) (Table 3). In contrast, no patients in Group B died.

**TABLE 3:** Details of death

	Group A	Group B
Desmoid tumors	3	0
Colon cancer	1	0
Gastric cancer	1	0
Sepsis	1	0

*Note:* Group A, patients who received colectomy; Group B, patients who did not receive colectomy.

## 4 DISCUSSION

This multicenter study investigated the current clinical practice for sparse and attenuated type FAP in Japan. Our findings highlighted four clinically important changes in the current clinical practice for sparse and attenuated type of FAP in Japan.

First, the current clinical practice for sparse and attenuated type of FAP and treatment strategies according to polyp density were investigated. Our results demonstrated that one‐third to one‐half of patients with sparse and attenuated type FAP did not receive colectomy in any age group (Figure 2a, b). This fact applies to the patients over 30 years of age, for whom colectomy is recommended in the JSCCR Guidelines.^7^ Furthermore, patients with attenuated type FAP have fewer polyps and the development of colorectal cancer at <30 years of age is rare; thus, this study further investigated the polyp density in Groups A and B (Figure 3a, b).^16^, ^17^ We found that the polyp density did not differ to a statistically significant extent between two groups in almost all of the age groups. That is, non‐colectomy management was proposed for patients in whom the polyp density was classified as sparse and attenuated. This is important information for determining the indication of non‐colectomy management.

Second, the main concern in relation to the selection of non‐colectomy management for FAP patients was the development of colorectal cancer that cannot be controlled by colonoscopy.^18^ Our results demonstrated that Group B had a significantly lower rate of patients who were lost to follow‐up than Group A (34% vs 20%, *P* = .003; Table 1), and although patients in Group B developed colorectal cancer, most patients (88%) were diagnosed at the Tis stage. This fact suggests that Group B received intensive endoscopic follow‐up, which could prevent the development of colorectal cancer that could not be controlled by colonoscopy during the intermediate follow‐up period (median age at the diagnosis of FAP, 24 years; follow‐up period, 13 years). Longer term follow‐up is required to determine whether these patients require colectomy during their lifetime.

Third, the type of surveillance that was performed for patients managed without colectomy would be a matter of interest. The interval of colonoscopy was basically determined by each institution and each physician, and one study answered this question in detail. Ishikawa et al demonstrated non‐colectomy management of FAP patients who refused colectomy, and some of their patients overlapped with patients from our study.^14^ They reported that 90 patients with sparse and attenuated type FAP received intensive endoscopic follow‐up in order to avoid colectomy. Consequently, colectomy could be avoided in all patients, with the exception of two patients whose condition progressed to profuse type FAP during the median follow‐up period of 5.1 years. Five patients were diagnosed with colorectal cancer; however, all were treated by endoscopic polypectomy. During this period, a median of eight endoscopic polypectomy procedures was performed per patient and a total 475 polyps/person were removed. This means that more polypectomy of >50 polyps was performed every 6 months for each patient. Thus, non‐colectomy management required specialized facilities and skilled practitioners. Despite these challenges, they concluded that it is possible to safely extend the timing of colectomy for patient with sparse and attenuated type FAP.

Fourth, long‐term survival was compared between two groups. No patients in Group B died, while six patients in Group A died, including three patients with desmoid tumors and one patient with colorectal cancer. This study focused on patients without colorectal cancer who required colectomy for the purpose of cancer removal. Thus, the colorectal cancer‐related mortality rate was 0.4% (1/250). On the other hand, desmoid tumor was the leading cause of death. The risk factors for the development of desmoid tumors were investigated by Saito et al using the same database, and female sex (odds: 2.2), colectomy at <30 years of age (odds: 1.9), prophylactic colectomy (odds: 1.9), and ileoanal anastomosis (IAA) (odds: 2.2) were identified, and the incidence of patients with these factors was approximately 20%.^19^ One notable fact is that the incidence of desmoid tumor development increases in female patients who undergo colectomy at a young age. Thus, Durno et al concluded that delayed colectomy should be considered for young female patients with FAP in order to reduce the chances of developing desmoid tumors.^20^ Thus, our clinical outcomes will contribute to the management of patients who are better suited to delayed colectomy.

Fifth, regarding concomitant lesions, the incidence of papillary adenoma in Group A was significantly higher than that in Group B, while the incidence of gastric adenoma in Group B was significantly higher than that in Group A (Table 2). In previous studies, papillary adenoma was detected in 29%‐72% of patients with FAP, and showed slow clinical progression.^21^, ^22^ Singh et al demonstrated that the incidence of the development of papillary adenocarcinoma was 1.4% during 7.8 years of follow‐up, and the risk factors for cancerization included male sex, abnormal appearance of papilla, prior cholecystectomy, and history of extracolonic malignancies.^23^ On the other hand, gastric adenoma develops in 9%‐50% of FAP patients, and it has been suggested that in Asian countries patients with gastric adenoma are have an increased risk of developing gastric cancer.^24^ Martin et al recommended endoscopic treatment for gastric adenomas of >20 mm in size, which are associated with an increased risk of high‐grade dysplasia.^25^ These facts were clarified by recent epidemiological studies; however, the impact of surgical intervention on the incidence of papillary adenoma and gastric adenoma were not well‐investigated and further studies are required.

Finally, looking at the trends in Europe and the United States, surgical treatment for FAP has shifted toward individualized treatments according to the localization and density of polyps, and several clinical studies are underway. One clinical study from Europe is investigating a personalized surveillance and intervention protocol for patients with FAP who have undergone (procto)colectomy (ClinicalTrials.gov Identifier: NCT04678011). After IRA or IPAA, a 1 to 2 yearly endoscopic surveillance interval is uniformly recommended for all patients, and no advice is provided on optimal surveillance for individual patients.^26^ Thus; researchers investigated optimal personalized endoscopic surveillance for individual patients who underwent IRA and IPAA. Another clinical study from the United States is investigating the non‐surgical management of non‐dense FAP patients (ClinicalTrials.gov Identifier: NCT02747862). The setting of the study was similar to our own, and they compared the clinical features between patients managed with and without colectomy. Thus, the strong point of this study is that the intermediate term outcomes (median 13 years after the diagnosis of FAP) of non‐colectomy management for FAP are presented in a nationwide multicenter study.

The present study was associated with some limitations. First, there were many missing data; however, these data could not be investigated further because it was impossible to link data to patients as their privacy was protected, and further investigations regarding missing data were impossible for this reason. Second, this was a retrospective study with a limited study population; however, it was nationwide multicenter study. Third, our database included three choices for the treatment status of FAP patients: cancer excision, prophylactic colectomy, and non‐surgical management. However, this information was missing for 128 patients, who were therefore excluded from the analysis, which could have been a source of bias. Fourth, it would be useful to clarify the patients who have to receive colectomy unintentionally due to reasons such as polypectomy trouble, change to profuse type, colorectal cancer that could not be controlled by endoscopic polypectomy, or other reasons. However, these details were not included in this database. Furthermore, our database did not include detailed data on surveillance in patients who received non‐colectomy management. Especially, the endoscopic follow‐up interval between subsequent colonoscopies for polyp removal in each institution are interesting; however, all questions were fixed in the first research plan and it was not listed in the question sheet. Addition, aspirin and cyclooxygenase‐2 inhibitors have been shown to reduce the development of colorectal tumors; however, this point is beyond the scope of this study.

In conclusion, the JSCCR Guidelines for FAP recommend total proctocolectomy for patients in their 20s; however, over one‐third of patients with non‐dense FAP in Japan did not receive colectomy at >30 years of age.^7^ Polyp density is an important factor when considering non‐colectomy management for FAP patients; however, our results demonstrated that non‐colectomy management was proposed for patients in whom the polyp density was classified as sparse and attenuated. Regarding survival, even though colorectal cancer developed in patients managed without surgery, most patients were diagnosed at the Tis stage, and the development of desmoid tumors was rare, which resulted in good survival in the non‐colectomy group, indicating that management without colectomy represents a potential option for the management of selected non‐dense FAP patients. However, non‐colectomy management requires intensive endoscopic follow‐up. Furthermore, the follow‐up period of this study was 13 years, and the results of longer‐term follow‐up are awaited to determine whether these patients require colectomy during their lifetime.

## DISCLOSURE

Funding: None.

Conflict of interest: Dr Yoji Takeuchi has received lecture fees from Olympus, Boston Scientific (Japan), Daiichi‐Sankyo, Miyarisan Pharmaceutical, Asuka Pharmaceutical, AstraZeneca, EA Pharma, Zeria Pharmaceutical, Fujifilm, Kaneka Medix, Kyorin Pharmaceutical, and the Japan Gastroenterological Endoscopy Society Ethics Statements. Dr. Hideki Ueno is an editorial member of *Annals of Gastroenterological Surgery*.

Ethics: The present study was approved by the ethical committees of the JSCCR (90–5) and Hyogo College of Medicine (3943).

Author contributions: Substantial contributions to the conception or design of the work, or the acquisition, analysis, or interpretation of data for the work: Takaaki Matsubara, Naohito Beppu. Drafting the work or revising it critically for important intellectual content: Masataka Ikeda, Hideki Ishikawa, Yoji Takeuchi, Toshiya Nagasaki, Akinari Takao, Kazuhito Sasaki, Kiwamu Akagi, Tomoya Sudo, Hideki Ueno. Final approval of the version to be published: Tatsuro Yamaguchi, Naohiro Tomita, Yoichi Ajioka.
